# Muscle Tone Physiology and Abnormalities

**DOI:** 10.3390/toxins13040282

**Published:** 2021-04-16

**Authors:** Jacky Ganguly, Dinkar Kulshreshtha, Mohammed Almotiri, Mandar Jog

**Affiliations:** London Movement Disorder Centre, London Health Sciences Centre, University of Western Ontario, London, ON N6A5A5, Canada; jganguly@uwo.ca (J.G.); Dinkar.Kulshreshtha@lhsc.on.ca (D.K.); Mohammed.Almotiri@lhsc.on.ca (M.A.)

**Keywords:** spasticity, rigidity, dystonia, paratonia

## Abstract

The simple definition of tone as the resistance to passive stretch is physiologically a complex interlaced network encompassing neural circuits in the brain, spinal cord, and muscle spindle. Disorders of muscle tone can arise from dysfunction in these pathways and manifest as hypertonia or hypotonia. The loss of supraspinal control mechanisms gives rise to hypertonia, resulting in spasticity or rigidity. On the other hand, dystonia and paratonia also manifest as abnormalities of muscle tone, but arise more due to the network dysfunction between the basal ganglia and the thalamo-cerebello-cortical connections. In this review, we have discussed the normal homeostatic mechanisms maintaining tone and the pathophysiology of spasticity and rigidity with its anatomical correlates. Thereafter, we have also highlighted the phenomenon of network dysfunction, cortical disinhibition, and neuroplastic alterations giving rise to dystonia and paratonia.

## 1. Introduction

Muscle tone is a complex and dynamic state, resulting from hierarchical and reciprocal anatomical connectivity. It is regulated by its input and output systems and has critical interplay with power and task performance requirements. Tone is basically a construct of motor control, upon which power is intrinsically balanced. This hierarchy of motor control includes cortex (extensive processing capability with highest degree of freedom), basal ganglia (learning and teaching of context dependent tasks with less degrees of freedom), cerebellum (fine-tuning), brainstem reticular system (common pathway for ascending and descending tracts), spinal cord (the main pathway for ascending and descending tracts), and muscle spindle (final common pathway with least degree of freedom). In this review, we have discussed the controversies regarding the definition of muscle tone and its classification, followed by the mechanisms and pathways responsible for maintaining tone. Spasticity and rigidity, the two types of hypertonia, have been elaborated in the context of the dysfunction in the supraspinal pathways and the interaction between spinal cord and muscle spindle. The other two disorders of altered tone, namely dystonia and paratonia, are not exactly related to the physiological dysfunction of the tone pathways. In the motor control system, spasticity and rigidity are predominantly an output system problem, while dystonia is a system level processing problem. Dystonia and paratonia have altered tone secondary to network disruption in the basal ganglia, the thalamocortical circuits, and their connections. The mechanisms underlying these have been discussed thereafter because they are important both clinically and pathophysiologically from a movement disorder perspective.

## 2. Definition of Muscle Tone

Muscle tone is traditionally defined as ‘the tension in the relaxed muscle’ or ‘the resistance, felt by the examiner during passive stretching of a joint when the muscles are at rest’ [1]. This definition of tone has some ambiguities such as, what does the ‘resistance to passive stretch’ mean is not clear and ‘felt by the examiner’ opens the door to subjective variation during clinical examination and interrater variability of the assessment [2]. Studies with electromyographic (EMG) assessment often equate muscle tone with baseline EMG level in a relaxed state. However, apart from the active or contractile component resulting from the activation of motor unit and detectable by EMG, muscle tone also has a passive or viscoelastic component, independent of neural activity that can’t be detected by EMG. The viscoelastic component in turn depends upon multiple factors like the sarcomeric actin-myosin cross-bridges, the viscosity, elasticity, and extensibility of the contractile filaments, filamentous connection of the sarcomeric non-contractile proteins (e.g., desmin, titin), osmotic pressure of the cells, and also on the surrounding connective tissues [3,4].

Mathematically, muscle tone can be interpreted as the change in resistance or force per unit change in length (Δ force/Δ displacement of the tissue) [5]. In a relaxed state, resistance to an external motion (R_TOT_) depends on inertia (R_IN_), apparent stiffness (resistance to stretch/R_ST_) and damping (resistance to velocity/R_DA_): R_TOT_ = R_IN_ + R_DA_ + R_ST_ [6]. However, all these definitions have a common fallacy of assuming that the person is in completely relaxed state, which is often impossible to achieve unless using muscle relaxants.

In contrast to this general notion, Bernstein highlighted the fact that muscle tone may actually reflect a state of preparedness to a movement and thus it may not be possible to estimate muscle tone when the person is asked to relax and not to make any movement [7]. Bernstein, in his hierarchical model of movement construction (tonus, synergies, space, action), postulated that muscle tone is an adaptive function of the neuromotor apparatus that responds adequately to commands coming from upper levels of movement construction by fine-tuning the excitability of the sensory and motor cells for the tasks of active postural or movement control [6,7]. This definition makes muscle tone an active contributor to movement and postural tasks. Similarly, Carpenter et al. have given a clinical definition of tone as “the constant muscular activity that is necessary as a background to actual movement in order to maintain the basic attitude of the body, particularly against the force of gravity” [8]. Thus, tone can be a construct that is required for motor control such that both static and dynamic tasks can be safely performed in the most thermodynamically efficient way. 

## 3. Classification of Muscle Tone

Muscle tone can be classified as ‘postural’ and ‘phasic’ types. Postural tone is seen in axial muscles where gravity is the most important inciting factor. It results from a steady stretch on the muscles and tendons and manifests as prolonged muscle contraction. In contrast, phasic tone is what is commonly assessed clinically in the extremities as a rapid and short duration response. It results from the rapid stretching of a tendon and attached muscle and more precisely, the muscle spindle [9]. Apart from this, muscle tone can be classified into its active and passive components, as has already been described above. 

## 4. The Anatomy Underlying the Regulation of Muscle Tone

Muscle tone is regulated by spinal and supraspinal mechanisms. While spinal control depends on the interaction between muscle spindle and spinal cord along with the interneurons, supraspinal control is regulated by facilitatory and inhibitory long tracts and cerebellum.

### 4.1. Spinal Control 

#### 4.1.1. Interaction between Muscle Spindle and Spinal Cord

Sensory feedback to spinal cord from the muscle, regarding its length and tension, is necessary for regulation of muscle tone. Intrafusal fibers send information of muscle length or rate of change of length, while Golgi tendon organs send information about tendon tension or rate of change of tension [10]. Type Ia afferents detect the velocity of the change in muscle length during a stretch (dynamic response). However, tonic activity of type Ia and II afferents detect steady-state length of the muscle (static response). Type Ib afferents send information from Golgi tendon organs. 

Muscle spindle generates tone by activating the stretch reflex. When a motor command is sent to the alpha motor fibers (supplying extrafusal fibers), gamma fibers (supplying intrafusal fibers) will also be excited (alpha–gamma co-activation), resulting in contraction of both extrafusal and intrafusal fibers [8,10]. Stretch reflex can be of two types: (a) Dynamic and (b) Static. Sudden rapid stretch of a muscle stimulates nuclear bag fibers (respond to rate or velocity of the stretch) and Ia afferents (annulospiral endings) carry the dynamic signal to spinal cord. Efferent signal from the cord (alpha motor neuron) comes via alpha efferents to extrafusal fibers, resulting in sudden contraction of the muscle (dynamic stretch reflex). This is the basis of clinical elicitation of the deep tendon reflexes. On the other hand, sustained stretch of the muscle stimulates nuclear chain fibers and type II afferents (flower-spray endings) carry the signal to cord. Efferent signal from the cord travels via alpha efferents to extrafusal fibers. However, this time there will be asynchronous contraction of the extrafusal muscle fibers (motor units not discharging all together) that will result in mild sustained contraction of these fibers as long as it is stretched. This static stretch reflex response is the physiological basis of maintaining muscle tone [8,10,11].

On the other hand, there is a threshold, when the stronger a muscle is stretched, stronger is the reflex contraction. After crossing the threshold, contraction stops, and the muscle relaxes. This is known as ‘inverse stretch reflex’ and is mediated by the Golgi tendon organ present in the fascicles of a tendon [11]. 

#### 4.1.2. Interneurons

**Figure 1:**
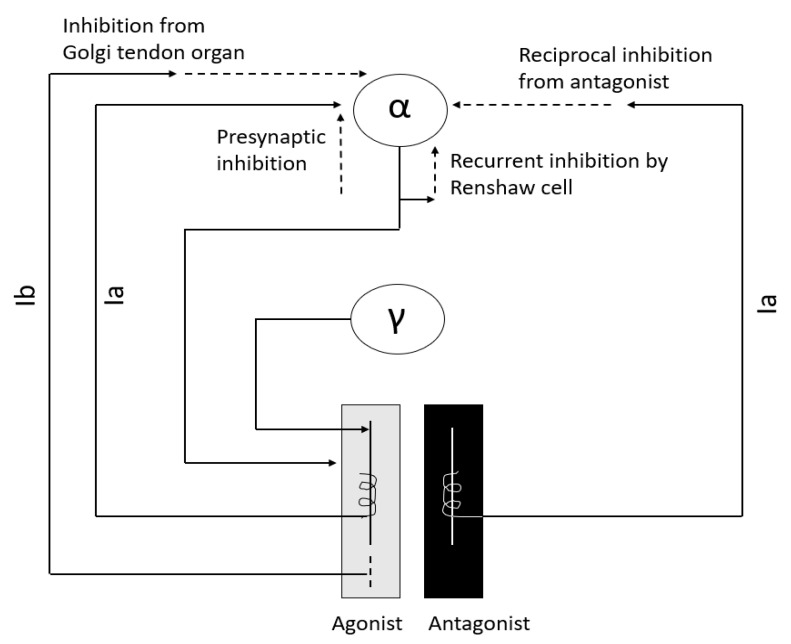
Major interneurons in regulation of muscle tone.

Interneurons are an integral part of the stretch reflex arc (Figure 1) and play a major role in maintaining muscle tone. They are inhibited or excited by multiple descending fiber systems. Multiple interneuronal pathways exist and their role in spasticity is discussed later. Among them, recurrent inhibition by Renshaw cell, reciprocal Ia inhibition from antagonist muscle, non-reciprocal Ib inhibition by Golgi tendon organ, and presynaptic inhibition are most important for maintenance of muscle tone [10,11,12,13,14].

### 4.2. Supraspinal Control via Descending Long Tracts 

**Figure 2:**
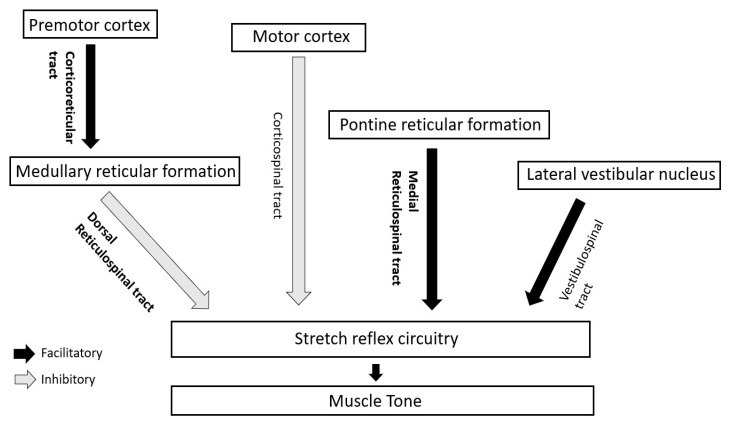
Descending long tracts in regulation of stretch reflex circuitry and muscle tone in humans. Main tracts for tone regulation have been highlighted in bold.

In human beings, the supraspinal impact on muscle tone and stretch reflexes is mainly modulated by the interplay (discussed later) of two inhibitory and two facilitatory descending tracts [10] (Figure 2).

#### 4.2.1. Inhibitory Tracts

- Corticospinal tract/CST (from motor cortex)
- Corticoreticular (from premotor cortex) and dorsal reticulospinal tract/ dorsal RST (from medullary reticular formation)

#### 4.2.2. Facilitatory Tracts

- Vestibulospinal tract/VST (from lateral vestibular or Deiter’s nucleus)
- Medial reticulospinal tract/medial RST (mainly from pontine reticular formation)

Among these tracts, muscle tone is mainly regulated by the inhibitory dorsal RST and facilitatory medial RST.

### 4.3. Role of Cerebellum

Medial part of anterior lobe of cerebellum activates medullary reticular formation from where dorsal RST arises. Hence, the area of cerebellum inhibits muscle tone indirectly through inhibition of gamma motor neurons via dorsal RST. However, lateral part of anterior lobe activates the pontine reticular formation. Therefore, it facilitates muscle tone indirectly via medial RST by stimulating gamma motor neurons [15,16]. In humans, because lateral part of anterior cerebellum is more developed, cerebellar lesions commonly produce hypotonia. On the other hand, vestibulocerebellum is connected with the vestibular nucleus that stimulates alpha motor neurons. Thus, the cerebellum is also an important regulatory site for ‘alpha-gamma linkage’ [17].

## 5. Spasticity

J. W. Lance in 1980 defined spasticity as “a motor disorder characterized by a velocity dependent increase in tonic stretch reflexes (muscle tone) with exaggerated tendon jerks, resulting from hyperexcitability of the stretch reflex, as one component of the upper motor neuron syndrome” [18]. However, apart from velocity, spasticity also depends on length of the muscle. Spasticity in the knee extensor (quadriceps) is more when the muscle is short, but in upper limb flexors (e.g., biceps) and ankle extensors (gastrocnemius, soleus), spasticity is more when the muscles are long. Lance’s definition also ignores the role of sensory input (discussed later) in spasticity. In 2005, the Support Program for Assembly of a Database for Spasticity Measurement (SPASM) project redefined spasticity as “disordered sensory-motor control, resulting from an upper motor neuron lesion, presenting as intermittent or sustained involuntary activation of muscles” [19]. The definition incorporated the role of defective sensory input (not only motor) in spasticity. Recently in 2018, IAB-Interdisciplinary Working Group for Movement Disorders defined spasticity in a broader sense as “involuntary muscle hyperactivity in the presence of central paresis” [20]. In this definition, the ‘involuntary muscle hyperactivity’ has been described as a spectrum consisting of—(i) ‘Spasticity Sensu Strictu’ triggered by rapid passive joint movements, (ii) ‘rigidity’ triggered by slow passive joint movements, (iii) ‘dystonia’ when the involuntary muscle hyperactivity is spontaneous and (iv) ‘spasms’ triggered by sensory or acoustic stimuli. The group has also proposed axis-based approach to spasticity—clinical description (axis 1), etiology (axis 2), localization (axis 3) and additional central nervous system deficits (axis 4) [20]. Severity of the muscle hyperactivity can be described by the Modified Ashworth Scale, Tardieu Scale and Frequency of Spasms Score.

Spasticity can be classified as ‘phasic’ and ‘tonic’ based on the predominant involvement of either the phasic (dynamic) or tonic (static) components of muscle stretch reflexes. After spinal injury, ‘phasic’ spasticity with brisk stretch reflexes and clonus develops in patients who are ambulatory. However, ‘tonic’ spasticity develops in non-ambulatory patients, demonstrated by passive stretch at ankle and vibratory tonic reflex testing [10,21].

In the following section, we will discuss spinal and supraspinal factors contributing to spasticity including the role of sensory feedback as evidenced by recent studies, followed by pathophysiology of clasp-knife phenomenon and clonus.

## 6. Factors Contributing to Spasticity

### 6.1. Spinal Influence

Spinal influence on spasticity can either be from increased excitation or decreased inhibition. Enhanced fusimotor drive [22], denervation hypersensitivity [23], axonal sprouting [24,25], hyperexcitability of alpha motor neurons [25], excitation of interneurons [25] and increased cutaneous stretch reflexes [10] are responsible for increased excitatory influence on spasticity. Animal models have shown the role of membrane properties of the motor neuron in spasticity. Voltage dependent persistent inward current (PIC) mediated via Na^+^ and Ca^2+^ channels can cause prolonged depolarization (plateau potential), modulated by the descending serotonergic and noradrenergic drive [10,25]. Descending monoaminergic drive normally has an excitatory effect on alpha motor neurons at the ventral horn via 5HT2 and NEα1 receptors, whereas it has inhibitory effect at dorsal horn via 5HT1b/d and NEα2 receptors [25]. In the acute stage after spinal injury, due to loss this descending monoaminergic influence, hypoexcitability of motor neurons occurs at the ventral horn whereas disinhibition and excitation of sensory input at the dorsal horn. However, spasticity doesn’t develop acutely despite interneuronal excitability until motor neurons regain the excitability. In the chronic stage, denervation hypersensitivity of ventral horn motor neurons occurs to the remaining monoaminergic input and PIC is activated that in turn leads to development of spasticity.

Apart from excitatory mechanisms contributing to spasticity, the role of altered spinal inhibitory circuitry is increasingly being appreciated in recent studies [10]. Disinhibition of the alpha motor neuron in spasticity can occur from decreased presynaptic inhibition of Ia afferents [26], decreased disynaptic reciprocal Ia afferent inhibition from the antagonist muscle group [27,28], decreased Ib afferent mediated inhibition by Golgi tendon organ [29], and altered recurrent inhibition by Renshaw cells (doubtful role) [30].

### 6.2. Supraspinal Influence

In the normal scenario, human muscle tone is critically balanced by the inhibitory drive of CST and dorsal RST and facilitatory drive (on extensor tone) by medial RST and to some extent VST [10]. Among them, dorsal RST also inhibits flexor reflex afferents (FRA). In the spinal cord, lateral funiculus contains the corticospinal tract (CST) and dorsal RST while anterior funiculus contains VST and medial RST. Based on this, the effects of cortical and spinal lesions on muscle tone can be summarized as follows.

#### 6.2.1. Cortical Lesions

Isolated involvement of CST is insufficient to produce spasticity [31,32]. Cortical lesions produce spasticity due to associated involvement of corticoreticular fibers, the connection between premotor cortex and medullary reticular formation from where dorsal RST originates. Hemiplegia with spasticity and antigravity posturing occurs because of unopposed facilitatory action of medial RST, in the absence of inhibitory influence of dorsal RST.

#### 6.2.2. Spinal Cord Lesions

- Incomplete/partial myelopathy involving lateral funiculus: If there is involvement of CST only, it will result in weakness, hypotonia and loss of superficial reflexes. If there is additional involvement of dorsal RST, spasticity and hyperreflexia will develop due to unopposed activity of medial RST. Spasticity will be predominant in antigravity muscles and will result in paraplegia in extension and extensor spasms. Flexor spasms can occur if FRA are activated by pressure sores. On the other hand, if there is involvement of dorsal RST only with sparing CST, there will be spasticity without much weakness.
- Complete myelopathy with involvement of all four tracts: Spasticity will be less in this case because of lack of facilitatory input from medial RST and VST. Disinhibition of FRA will result in paraplegia in flexion and flexor spasms.

### 6.3. Role of Sensory Feedback

Recent studies have argued that co-activation of antagonist muscles, stiff posture and stiff gait of spasticity may be the adaptations to stabilize the joint and posture in a background of decreased muscle strength, whereas hyperexcitable reflexes play a minor or no role [33,34]. Spastic movement disorders may result due to inadequate prediction of the sensory consequences of movements [35,36]. Due to the absence of a firm prediction of somatosensory feedback from the moving limb, the patient with UMN lesion will have difficulty in optimizing the movement. Co-contraction of the muscles around the joint may therefore be a strategy to minimize random movement and to stabilize the movement as far possible [33]. Therefore, spastic movement disorder may rather be a compensation to the weakness. The concept can be implemented in uncomplicated hereditary spastic paraplegia (HSP), where large fiber proprioceptive sensory loss occurs along with corticospinal tract (CST) involvement. As discussed previously, selective loss of CST is not sufficient to produce significant spasticity more than weakness. Thus, defective sensory feedback may also play a role in the clinical manifestation of marked spasticity out of proportion to weakness in HSP. DeLuca et al. highlighted that, in HSP, axonal loss occurs in both large (>3 µm^2^) and small (<3 µm^2^) diameter nerve fibers of motor (CST) and sensory (posterior column) tracts, whereas in multiple sclerosis (MS) small diameter fibers are preferentially affected [37]. Thus, the affection of large diameter nerve fibers may be responsible for the predominance of spasticity seen in HSP, in comparison to MS where weakness predominates.

### 6.4. Non-Neural Factors

As discussed previously, changes in non-neural factors like tissue viscoelastic properties (e.g., elastic stiffness, viscous damping) can also contribute to the generation of spasticity [38,39].

## 7. Pathophysiology of Clasp-Knife Phenomenon

While passively stretching a muscle, more resistance is felt in the initial part of stretching, but with continued stretching, a sudden release of resistance occurs, described as ‘clasp-knife phenomenon’. Because of length dependency of spasticity, initially while bending the knee (quadriceps is short) increased resistance is felt (spasticity is more). However, with continued stretching (quadriceps is lengthening), after reaching a critical length, the resistance suddenly decreases [40,41,42]. The excitation of slowly conducting, higher-threshold inhibitory group III and IV muscle afferents (part of flexor reflex afferents/FRA) may also be responsible for this phenomenon [43].

## 8. Pathophysiology of Clonus

Clonus is defined as the “regular, repetitive, rhythmic contractions of a muscle subjected to sudden, maintained stretch” [11]. Clonus sustained for five or more beats is considered as clinically abnormal. The pathological basis has been explained in the literature in multiple ways: (1) stretch reflex-inverse stretch reflex sequence, (2) disruption of the Renshaw cell and type Ia inhibitory interneuron mediated inhibition of the antagonist → repetitive sequential contraction of agonist and antagonist → clonus results, (3) hyperactivity of the muscle spindles → activation of all the motor neurons from the burst of impulses coming from the spindle → consequent muscle contraction stops spindle discharge → during maintenance of the sustained stretch, the muscle is again stretched as soon as the muscle relaxes → spindles are again stimulated [11,44]. However, the exact pathophysiology is still debated.

## 9. Rigidity

Rigidity, in contrast to spasticity, doesn’t depend on the velocity of movement. It equally affects flexors and extensors and gives rise to uniform resistance to passive stretching in all directions known as ‘lead pipe’ phenomenon (Table 1). Hypertonicity in PD was also noted to be regularly interrupted as a ‘cogwheel phenomenon’ at a 6–9 Hz frequency, that is higher than the frequency of rest tremor (4–5 Hz) and postural tremor (5–6 Hz) [45,46]. As per recent consensus, superimposed tremor, or “an underlying, not yet visible, tremor” [47] results in intermittent increase in tone during the passive movement of a joint and gives rise to ‘cog wheel’ rigidity in PD [48]. Thus, cog-wheeling can be present even if there is no overt tremor [49]. Rigidity is one of the cardinal signs of PD. It is present in both the phenotypes of PD (‘akinetic-rigid’ and ‘tremor dominant’), while more marked in the former phenotype. While appendicular rigidity is generally more than axial rigidity in idiopathic Parkinson’s disease (IPD), marked axial rigidity indicates atypical parkinsonism like progressive supranuclear palsy (PSP) [50].

In the following section, we will discuss factors contributing to rigidity followed by pathophysiology of activation maneuver (Froment’s maneuver).

## 10. Factors Contributing to Rigidity

### 10.1. Exaggeration of Long-Latency Stretch Reflexes (LLSR)

Initial studies suggested that parkinsonian rigidity is likely of spinal reflex origin, substantiated by the fact that the rigidity improved by dorsal cord resection [51]. Enhanced response of the muscle receptors to passive stretch was thought to be the main culprit. However, subsequent studies with microneurographic recordings found that the increased muscle afferent discharge from enhanced fusimotor drive was not sufficient enough to cause rigidity [52]. Monosynaptic segmental stretch reflexes didn’t differ significantly between PD patients and healthy subjects in the studies utilizing electrophysiological analysis [53,54]. Rather, Ia afferent mediated spinal reflexes like tendon jerks, H-reflex and tonic vibration reflex were found to be mostly normal in PD patients [55,56]. So, the notion gradually shifted to the exaggerated long-loop or long-latency stretch reflex (supraspinal influence) on parkinsonian rigidity rather than spinally mediated reflexes. Berardelli et al. noted co-relation between increase of LLSR with rigidity and suspected the role of group II afferents in this regard [57]. Rothwell et al., although noted enhanced LLSR in PD, couldn’t find any quantitative correlation of this with rigidity. They suggested that LLSR is not solely responsible for parkinsonian rigidity and enhanced late polysynaptic reflexes mediated by cutaneous afferents may also be important [53].

### 10.2. Enhanced Shortening Reaction (SR) and Stretch-Induced Inhibition (SII)

Exaggerated LLSR can explain hypertonia in PD but cannot explain the resistance to passive stretch being uniform throughout the range of movement (‘lead pipe’ character)? Anomalous reaction in the muscle that is shortening was initially noted by Westphal and subsequently named ‘shortening reaction’ (SR) by Sherrington [58]. Alteration in the pathways for short-latency autogenic inhibition, mediated by altered excitability of Ia and Ib spinal interneurons, may be responsible for the phenomenon of SR [59]. On the other hand, a sudden decrease in resistance is seen while continuously stretching or lengthening a muscle beyond a critical joint angle. The phenomenon is called a ‘lengthening reaction’ or stretch-induced inhibition (SII) [60]. The combined effect of SR and SII generate ‘lead pipe’ effect while examining limb tone in PD [61].

### 10.3. Role of Brainstem

**Figure 3:**
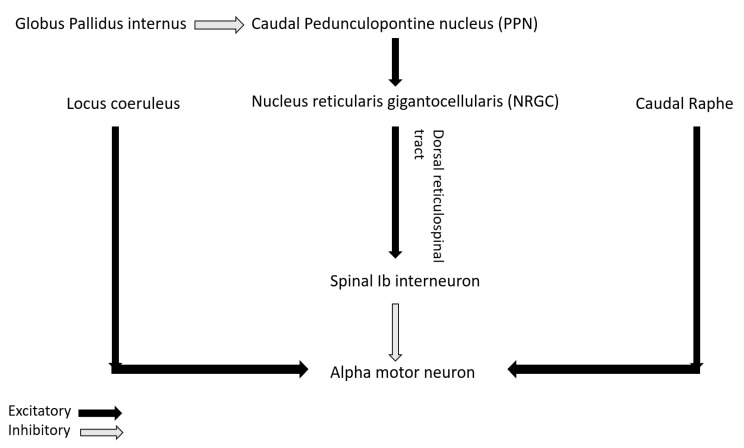
Neuromodulatory inputs in pathophysiology of rigidity.

The role of non-dopaminergic system in PD is increasingly being highlighted in recent studies. Recently, Linn-Evans et al. have noted increased and more symmetric rigidity in upper limb of PD patients in wakefulness who have REM sleep without atonia (PD-RSWA+) compared to with-atonia (PD-RSWA-) and controls [62]. The brainstem circuit responsible for tone in REM sleep overlaps with the circuit maintaining motor neuron excitability and postural control in wakefulness [63]. In PD, there is evidence of alpha-synuclein deposition in the nuclei of both the circuits including sublaterodorsal nucleus (responsible for tone regulation in REM sleep), nucleus reticularis gigantocellularis (NRGC), locus coeruleus, caudal raphe and pedunculopontine nucleus (PPN) [64]. Caudal PPN gives cholinergic excitatory inputs to NRGC from where dorsal or lateral reticulospinal tract (dorsal RST) originates and activates Ib spinal interneurons that in turn inhibits alpha motor neuron (Figure 3). PPN also receives inhibitory input from globus pallidus interna (GPi). In PD, degeneration of PPN and NRGC decrease excitation of Ib spinal interneurons that in turn disinhibit alpha motor neuron and can lead to rigidity [65]. Increased inhibitory tone from GPi to PPN in PD can also lead to the same phenomenon. Heckman et al. have also highlighted the role of noradrenergic and serotonergic influence from locus coeruleus and caudal raphe respectively on motor neuron excitability by promoting firing via persistent inward current (PIC) [66]. Affection of these pathways in PD may lead to altered firing pattern of the motor neuron in response to incoming inputs and can contribute to rigidity [62].

### 10.4. Non-Neural Factors

As discussed earlier, viscoelastic properties of muscle fiber and surrounding connective tissues may also contribute to parkinsonian rigidity. Watts et al. have noted that in PD patients even with mild motor symptoms, upper limb stiffness was more than controls in relaxed state, but without any EMG activity [67]. The study highlighted the role of passive mechanical properties for the stiffness. Xia et al. have also noted the contribution from both neural and non-neural factors for parkinsonian rigidity while neural contribution dominates [68].

### 10.5. Network Hypothesis of Parkinsonian Rigidity

Baradaran et al. [69] explored the alteration in functional connectivity in brain networks in relation to parkinsonian rigidity. With progression of rigidity, they noted progressive abnormality of premotor → pre-cuneus connection (disease related change), while cerebellar → premotor connection approached normal values (compensatory mechanism). Kann et al. [70] have postulated that significant loss of gray matter and aberrant functional connectivity in fronto-parietal networks (critical for motor planning and execution) in akinetic-rigid subtype of PD are responsible for more aggressive course of functional decline, compared to tremor-dominant subtype.

## 11. Pathophysiological Basis of Activation Maneuver

An interesting but not yet clearly understood phenomenon seen in PD is the increase of rigidity with muscle contraction (isometric/rhythmic) in contralateral limb. Possible explanations described in the literature for this phenomenon are—(1) Enhanced LLSR via transcortical pathway [71,72], (2) Altered spinal motoneuron excitability mediated by the crossed sensory afferent feedback via lb afferent input resulting in diminished shortening reaction (SR) and increased sensitivity to passive stretch [73], (3) Facilitation of bilateral descending reticulospinal projection [62] and (4) A ‘Jendrassik maneuver’ equivalent where muscle contraction in other body part facilitates the H-reflex and enhances stretch reflex response [73].

## 12. Dystonia

### 12.1. Pathophysiology and Mechanisms

Dystonia is defined as ‘sustained or intermittent muscle contractions resulting in abnormal, often repetitive, movements, postures, or both’ [74]. These movements are typically patterned, may be tremulous or twisting and aggravated by voluntary action [75]. Dystonia can be focal, segmental, restricted to one half of the body (hemi dystonia), or generalized. The heterogeneous presentation and the various aetiologies’ ranging from genetic causes to neurodegenerative disorders point to multiple mechanisms that contribute to the pathophysiology of dystonia. The hypothesis in discussing the mechanisms of dystonia has largely centred around the basal ganglia-thalamo-cortical circuitry. However, advanced imaging modalities and electrophysiological experiments have widened the neuroanatomic correlates contributing to dystonia. The phenomena of sensory tricks and mirror movements seen in dystonia point to abnormalities in sensory processing and sensori-motor integration.

### 12.2. Anatomical Correlates of Dystonia

Basal ganglia and its abnormal connections have been implicated as the most important structure associated with dystonia. Secondary dystonias due to basal ganglionic lesions are a telling example of this association for a long time [74,75]. Levodopa improving dopa responsive dystonias (DRD) or deep brain stimulation of the internal segment of the globus pallidus that improves some cases of dystonia are further pointers to the central role of basal ganglia in the origin of dystonia [76,77,78]. Dystonia is not frequently found in spinocerebellar ataxias (SCA), but rarely it is a presenting symptom where cerebellar atrophy is the prominent feature with sparing of the basal ganglia. SCA-6 has a pure cerebellar pathology and generally present as a pure cerebellar ataxia phenotype. However, dystonia can be seen in these patients and that cannot be explained by basal ganglia dysfunction [76]. Draganski et.al., on voxel-based studies, showed increased grey matter density in globus pallidus interna, motor cortex and cerebellar floculus [77]. Garraux et al. demonstrated a significant increase in the grey matter volume in the hand representation area of patients with focal hand dystonia using voxel-based morphometry studies. This peri-rolandic increase was also seen in dorsolateral prefrontal cortex, inferior parietal areas and cerebellum, but not in the lentiform nucleus [78]. Similar structural abnormalities in writer’s cramp have been noted in primary sensorimotor cortex, cerebellum and pulvinar nuclei of the thalamus [79].

Thus, it is quite clear that no single anatomical correlate is responsible for dystonia. Rather, it is the abnormal network connections between the basal ganglia, cerebellum, thalamus, and cortex that cause dystonia. In the following section, we will discuss the neurophysiological alterations postulated in this circuitry that gives rise to dystonia.

### 12.3. Mechanisms of Dystonia

In the absence of animal models of dystonia, the understanding of the pathophysiological mechanisms causing dystonia were brought to light with the advent of transcranial magnetic stimulation (TMS) and repetitive transcranial magnetic stimulation (rTMS). The TMS protocols developed as a research tool have highlighted the following physiological dysfunctions in patients with dystonia.

#### 12.3.1. Loss of Inhibition

Lack of surround inhibition, co-contraction of antagonistic muscles and overflow of activity into the muscles not intended for the action are all due to the physiological loss of inhibition [80,81]. Surround inhibition is a cortically driven phenomena where the muscles around an active contracting muscles are actively inhibited to prevent overflow contraction [81,82]. Sohn et. al. described this phenomenon in healthy volunteers using a single TMS pulse. On voluntarily flexing the index finger innervated by the median nerve, the motor evoked potential (MEP) recorded on TMS from the ulnar innervated abductor digiti minimi (ADM) was reduced [83]. The same author group demonstrated that in seven patients with focal hand dystonia compared to controls, the MEP amplitudes of ADM were enhanced by up to 270% during index finger flexion, thus, suggesting lack of surround inhibition [84]. Similarly, enhanced cortical excitability on TMS delivered at different stimulus intensities and with different levels of muscle facilitation was shown in 11 patients with task specific dystonia by Ikoma et.al., suggesting a higher number of excited motor units [85]. Electrophysiologically, intracortical inhibition is assessed by measuring the cortical silent period (CSP), the interruption of a voluntary electromyographic muscle activity following a supra-threshold TMS pulse. Rona et. al., found that the CSP was shorter in dystonia in ten patients with dystonia, more so in task specific dystonia than generalized dystonia [86]. In dystonia, the monosynaptic and long-latency stretch reflexes discussed earlier are prolonged with slower stretch and often evoke reflex activity in remote muscles, suggesting overflow [87]. Breakdown of reciprocal inhibition is responsible for the co-contraction of the opposing muscles, classically seen in dystonia. The spinal reflex abnormalities in dystonia are speculated to be due to the dysfunctional descending inputs from the higher centres [88].

#### 12.3.2. Abnormal Sensory Function

Pain is a well-recognized symptom of dystonia and has been reported in cervical dystonia in about 70% cases. In the absence of overt sensory abnormalities on clinical examination, these patients have sensory phenomena like photosensitivity in blepharospasm or neck pain prior to cervical dystonia [89]. The most fascinating phenomena in dystonia is the ‘geste antagoniste’ or the sensory trick. It is a voluntary manoeuvre performed by the patient to reduce the severity of the dystonia. The sensory trick alleviating the dystonia misidentified the disorder as being psychogenic in origin [90]. However, it was subsequently shown that abnormal sensory function and disordered sensorimotor integration is an important physiological alteration seen in dystonia. Hence, transcutaneous vibration to the muscle at a frequency of 50–120 Hz produces the tonic vibration reflex (TVR), a polysynaptic spinal cord reflex involving the muscle spindle afferents and gamma motor neurons. There is a reduced perception of TVR suggesting abnormal processing of the muscle spindle 1a afferents in idiopathic focal dystonia, not only across the symptomatic body parts but also in the unaffected regions [91]. There is a hypothesis that the sensory trick works by decreasing the gamma drive to the spindles relative to the alpha motor neuronal activity [92].

The grating orientation task (GOT) measures the acuity for spatial discrimination. It is administered on the fingertips of both hands and identifies the smallest grating ridge width for which the orientation can be accurately distinguished [93]. Numerous studies have identified GOT abnormalities with increased spatial discrimination threshold in patients with focal hand dystonia, blepharospasm and cervical dystonia [94,95,96]. The somatosensory temporal discrimination threshold (STDT) measures the temporal processing of sensory information and is the shortest interval at which two tactile stimuli delivered to the same body part can be temporally separately recognized [93]. Bradley et al. examined the STDT in thirty-five adult onset primary and forty-two unaffected first-degree relatives with voxel-based morphometry (VBM) was done to assess putaminal volumes in relatives with abnormal and normal TDTs. In thirty-two patients and twenty-two unaffected relatives, STDT was abnormal. VBM in thirteen unaffected members with abnormal STDTs and twenty unaffected members with normal STDTs showed bilateral increased grey matter volume in the putamen in those with abnormal STDTs. Authors concluded that the STDT reflects effective processing of the sensory stimuli by the putamen which is abnormal in patients with dystonia [97]. Thus, sensory dysfunction in dystonia involves not only the somatosensory processing but also peripheral sensory abnormalities as seen in GOT and TVR studies. Abnormalities in the processing of sensory information lead to a dysfunctional connectivity between the subcortical structures (basal ganglia, thalamus, superior colliculus) and primary somatosensory area (S1). The thalamus plays a pivotal role in integrating the sensory inputs with the basal ganglia and cerebellar outputs while the basal ganglia serve as a gatekeeper for sensory inputs. Abnormal sensorimotor integration thus plays a major role in the pathogenesis of dystonia.

#### 12.3.3. Abnormal Synaptic Plasticity

The abnormal sensory feedback and the loss of inhibition causes short and long-term changes in the cortical-subcortical circuits. Byl et al., performed experiments on monkeys to evaluate the role of repetitive strain injuries causing focal dystonia to highlight the neuroplasticity/learning origin of dystonia. They postulated that this degradation of sensory feedback in repetitive strain injury was a result of neuronal plasticity and substantially influenced the primary motor area giving rise to dystonia [98]. This phenomena of excessive magnitude of plasticity responses and unspecific topography of the spread (abnormal spread) has been demonstrated in human experiments as well and represents an important underlying mechanism of dystonia [99]. The importance of long-term potentiation (LTP) and long-term depression (LTD) in cortical plasticity are widely recognized and are believed to alter receptive sensory fields and motor representations in the brain. TMS experiments using paired associative stimulation (PAS), where low frequency median nerve stimulation and TMS are paired, probes synaptic changes in motor cortex [100]. In a case-control study on writer’s cramp patients, PAS was performed on the dominant hemisphere with electrical stimulation of the median or ulnar nerve combined with TMS delivered to the contralateral cortex 21.5 ms or 10 ms after the peripheral nerve stimulation. The motor response was measured from the abductor pollicis brevis (APB) and adductor digiti minimi (ADM) muscle. In control subjects, the motor potentials recorded after 21.5 ms increased in amplitude if the afferent PAS-component came from a homologous peripheral region. In patients with writers’ cramp however, either median or ulnar PAS at 21.5 ms increased the APB and ADM motor unit potential amplitudes. The increase in cases was higher in magnitude, started earlier, and was longer in duration. The authors concluded that that patients exhibited abnormal dynamic responses to PAS protocols suggesting neuronal plasticity of different polarities, consistent with LTP and LTD [101]. Similarly, PAS experiments by Quartarone et al. showed that in patients with dystonia, there was a stronger and a long lasting facilitatory increase in corticospinal excitability along with a loss of topographical specificity of PAS with facilitation in both the median and ulnar innervated muscles [102]. This experiment further reiterates the fact that overflow phenomena or co-contraction can be related to abnormalities of spatial properties of associative plasticity. Dystonia may thus be caused by an abnormal excessive association between sensory inputs and motor outputs. However, a recent study has highlighted that in contrast to primary dystonia, reduced somatosensory inhibition and enhanced cortical plasticity may not be required for the clinical expression of secondary dystonia [103].

### 12.4. Mirror Movements in Dystonia

Mirror movements represent an expression of motor overflow in dystonic patients. It is defined as the appearance of abnormal posturing in the affected limb induced when the contralateral unaffected limb is engaged in a specific task [104]. This helps differentiate between true dystonic movements or any compensatory movements done involuntarily to reduce the disability. Interhemispheric inhibition has been demonstrated on functional neuroimaging and electrophysiological studies, and its loss has been implicated in mirror movements. Merello et al. demonstrated on functional MRI that in a patient with writer’s cramp and mirror movements, on writing with the affected hand, there was contralateral and ipsilateral activation of the posterior parietal cortex and putamen and ipsilateral activation of the inferior frontal gyrus. However, on writing with the unaffected hand, greater activation was seen ipsilaterally suggesting attenuation of the transcallosal inhibition and inhibitory intracortical circuits [105]. Beck et al. used TMS to show lack of interhemispheric inhibition of the dystonic motor cortex during the premotor phase, not seen in those without mirror movements [106].

### 12.5. Dystonia as a Network Disorder

**Figure 4:**
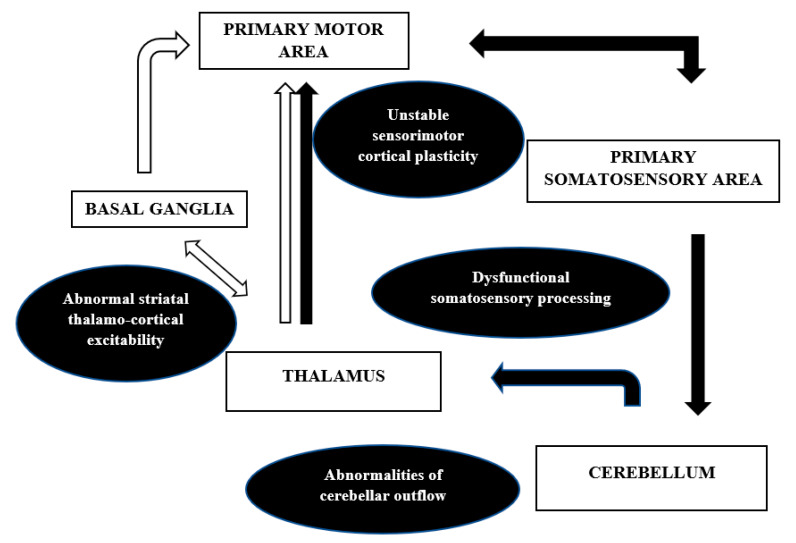
Network model of dystonia. White arrows: pallido-thalamo-cortical network, Black arrows: cerebello-thalamo-cortico-cerebellar network.

The anatomical correlates and the mechanisms discussed above are in contradiction to the earlier proposed notion of dystonia being a purely basal ganglia disorder. There is an increased recognition of the cortical-subcortical circuits, namely pallidothalamocortical and cerebello thalamocortical, in the pathophysiology of dystonia. This network model (Figure 4) can well explain the misprocessing of sensory information along with excitability of the inhibitory pathways at different levels of the neuroaxis that may result in abnormal plasticity and overt dystonia.

The network hypothesis also explains the focal onset and gradual segmental or generalized spread of the abnormality [107]. The basal ganglia and its thalamocortical connections through the direct and indirect pathway and the hyper direct pathway from the cortex to the subthalamic nucleus has been well described previously [87,108]. The direct pathway facilitates, while the indirect pathway inhibits movement. The hyperactivity of the direct pathway was demonstrated by Simonyan et al. in focal dystonias. The availability of the D1 receptors was significantly increased in putamen in patients with writer’s cramp and laryngeal dystonia, and this, adds support to the abnormal striato-thalamo-cortical excitability in dystonia [109]. Another example suggesting the network mechanism comes from the success of deep brain stimulation (DBS) of the internal segment of globus pallidus in dystonia. The DBS induced changes in the neuronal patterns inhibit the pathological bursts and oscillations in the network, resulting in better sensorimotor processing and symptomatic improvement [110]. The cerebellar connections in dystonia were studied by Argyelan et al. using magnetic diffusion tensor imaging and probabilistic tractography in DYT1 and DYT 6 mutation carriers. They showed reduced integrity of cerebellothalamocortical fibre tracts both in manifesting and non-manifesting mutation carriers, with greater deficits in the former group [111]. The authors concluded that the abnormalities of the cerebellar outflow may lead to the lack of cortical inhibition in dystonia. Both basal ganglia and cerebellum project to the SMA and pre-SMA, with a larger contribution of the basal ganglia pallidal neurons, as shown by Akkal et al. using retrograde trans neuronal transport of neurotropic virus in monkeys [112]. Thus, as per the network, it is not only the anatomical dysfunction in basal ganglia or thalamus that causes dystonia, but a functional disruption of the pathways discussed above that is important in the pathophysiology of dystonia.

Thus, recent experimental evidence has shifted our notion from dystonia being a psychiatric diagnosis to it being a disease characterized by alterations at different nodes in the brain. Basal ganglia are central to dystonia but other regions throughout the motor circuit contribute to its pathophysiology. The abnormalities in the networks, involving basal ganglia, cerebellum, thalamus, and cortex, are affected by abnormal sensory input, playing a key role in abnormal sensorimotor integration giving rise to dystonia.

## 13. Paratonia

Higher order motor disorders are the dysfunctional motor behaviours preceding voluntary execution of movement. These include disorders of disinhibition, motor intention, alien limb syndromes and mirror movements [113]. In the context of movement disorders, mirror movements have been discussed in the dystonia section. Alien limb phenomena and motor intention disorders are cortical phenomena well described in parkinsonism but beyond the scope of present discussion on disorders of tone. However, we would briefly like to discuss here a disorder of disinhibition, paratonia, as botulinum toxin is known to relieve the involuntary resistance seen in paratonia. Paratonia, first described by Friedlander in 1828, and later by Dupre in 1910, is described as increased muscle tone in response to passive movement, proportional to the strength of the stimulus applied. The degree of resistance depends upon the speed of movement. It can be faciliatory in the early stages, where the patient actively assists passive movements and becomes oppositional with advancing pathology when the resistance increases with increasing movements [113,114]. Unlike rigidity, paratonia is non-velocity dependent and the absence of a catch and the fact that paratonia can be elicited in any direction of movement differentiates it from spasticity [115].

## 14. Pathophysiology of Paratonia

Beversdorf et al. demonstrated that facilitatory paratonia was a form of echopraxia with the subjects mimicking examiner’s movements. They concluded that this is a form of defective response inhibition secondary to frontal lobe dysfunction. Orbitofrontal damage and frontal-subcortical dysfunction has been linked to defective response inhibition [116]. Goal directed movement is mediated by two circuits converging on the primary motor cortex: the pre-supplementary motor area (Pre-SMA)-primary motor and the parieto-premotor-primary motor area circuit. The former receives inputs from the prefrontal cortex and basal ganglia and modulates internally driven voluntary motor movements. The latter circuit is involved in object-oriented actions like grasping or following visual or verbal cues. The two circuits are normally balanced allowing a smooth execution of goal directed tasks. The pre-SMA normally inhibits the parieto-premotor circuit and this is disrupted in frontal lobe dysfunction. The result is a disinhibited behaviour and the individual depends on visual and tactile stimulation to drive motor movements with defective inhibition, contributing to facilitatory paratonia [113,117]. A peripheral mechanism has also been hypothesized contributing to the genesis of paratonia. The reciprocal inhibition at the spinal level is affected by advancing age and this causes a co-contraction of agonist and antagonist, perceived as increasing resistance to movement [114]. However, the electromyographic assessment of patients with paratonia did not reveal co-contraction of the opposing group of muscles in paratonia. Drenth et al. proposed another peripheral biochemical change that could contribute to increasing tissue resistance in patients with dementia and paratonia. Advanced glycation end products (AGEs), measured through skin autofluorescence were found to be significantly increased in patients with paratonia and had a direct relation to the severity of paratonia. AGEs increase tissue stiffness by forming cross links in muscle collagen, impair skeletal muscle function and may play a role in the pathogenesis of paratonia [118]. Basic pathophysiological mechanisms responsible for spasticity, rigidity, dystonia, and paratonia are highlighted in Table 2.

## 15. Conclusions

Tone is maintained by complex interplay of spinal and supraspinal mechanisms, disruptions of which lead to spasticity and rigidity. Altered tone can, however, be seen in dystonia and paratonia, disorders resulting due to network dysfunction, abnormal sensorimotor integration, and disinhibition in the brain and spinal cord. In a clinical scenario of hypertonia, differentiating these four disorders is of the utmost importance from pathophysiological and therapeutic perspectives.

## Figures and Tables

**Table 1:** Difference between spasticity and rigidity.

Differentiating Points	Spasticity	Rigidity
Velocity dependency	Yes	No
Resistance to movement	In one direction (flexion or extension)	In both directions
Length dependency	Yes	No
Type of hypertonicity	Clasp-knife	Lead pipe or Cog-wheel

**Table 2:** Pathophysiological basis of spasticity, rigidity, dystonia and paratonia.

Abnormalities in Tone	Basic Pathophysiology
Spasticity	Altered spinal excitatory and inhibitory circuitry leading to increased excitation and decreased inhibition Supraspinal influence predominantly involving inhibitory drive from dorsal reticulospinal tract (dorsal RST) and facilitatory drive from medial reticulospinal tract (medial RST) Abnormal sensory feedback Non-neural factors like viscoelastic properties of muscle fiber and surrounding connective tissues
Rigidity	Exaggeration of long-latency stretch reflexes (LLSR) Enhanced shortening reaction (SR) and stretch-induced inhibition (SII) Involvement of brainstem circuits involving sublaterodorsal nucleus, nucleus reticularis gigantocellularis (NRGC), locus coeruleus, caudal raphe and pedunculopontine nucleus (PPN) Alteration in functional connectivity in brain networks involving frontoparietal connection, premotor-pre-cuneus connection Non-neural factors like viscoelastic properties of muscle fiber and surrounding connective tissues
Dystonia	Lack of surround inhibition Abnormal sensory-motor integration Abnormal synaptic plasticity Abnormalities in pallido-thalamo-cortical and cerebello-thalamo-cortical network
Paratonia	Defective response inhibition from orbitofrontal damage and frontal-subcortical dysfunction Non-neural: Increase tissue stiffness from advanced glycation end products (AGE) deposition

## Data Availability

No new data were created or analyzed in this study. Data sharing is not applicable to this article.
